# Supplementary material for: Active site specificity profiling datasets of matrix metalloproteinases (MMPs) 1, 2, 3, 7, 8, 9, 12, 13 and 14
Source: Data Brief. 2016 Feb 22;7:299–310. doi: 10.1016/j.dib.2016.02.036 (PMC4777984; doi:10.1016/j.dib.2016.02.036)
Supplement: Supplementary file 10 — Supplementary material [file mmc10.zip › WebPICS_hMMP8_G_1%/P1.html]

 

PICS results


|  |  |
| --- | --- |
| **P1\_A**  13 in 120 sites   10.8 %    effects > 10 perc. pnts.  (vice-versa in brackets)  P3\_A: 26.2 (14.2)   P2\_A: 11.4 (10.6)   P2\_K: 29.3 (34.7)   P1prime\_Q: 20.8 (22.5)   P2prime\_K: 33.0 (17.2)   P3prime\_G: 29.6 (13.3) |  |
| **P1\_N**  23 in 120 sites   19.2 %    effects > 10 perc. pnts.  (vice-versa in brackets)  P3\_P: -19.0 (-15.6)   P2\_L: 12.8 (18.3)   P1prime\_F: 11.6 (37.9)   P2prime\_K: -12.1 (-11.2) |  |
| **P1\_P**  9 in 120 sites   7.5 %    effects > 10 perc. pnts.  (vice-versa in brackets)  P3\_H: 29.1 (52.5)   P2\_L: 20.0 (11.3)   P2\_W: 30.0 (67.5)   P1prime\_F: 27.5 (35.4)   P2prime\_Q: 34.4 (25.8)   P3prime\_V: 25.0 (22.5) |  |
| **P1\_Q**  11 in 120 sites   9.2 %    effects > 10 perc. pnts.  (vice-versa in brackets)  P2\_A: 15.6 (12.2)   P2\_F: 28.9 (35.2)   P1prime\_V: 17.3 (15.8) |  |
| **P1\_S**  14 in 120 sites   11.7 %    effects > 10 perc. pnts.  (vice-versa in brackets)  P2prime\_V: 15.3 (13.3) |  |
